# Prospective, Longitudinal Study on Specific Cellular Immune Responses after Vaccination with an Adjuvanted, Recombinant Zoster Vaccine in Kidney Transplant Recipients

**DOI:** 10.3390/vaccines10060844

**Published:** 2022-05-26

**Authors:** Monika Lindemann, Charleen Baumann, Benjamin Wilde, Anja Gäckler, Lara Meller, Peter A. Horn, Adalbert Krawczyk, Oliver Witzke

**Affiliations:** 1 Institute for Transfusion Medicine, University Hospital Essen, University Duisburg-Essen, 45147 Essen, Germany; baumann.charleen@gmail.com (C.B.); peter.horn@uk-essen.de (P.A.H.); 2 Department of Infectious Diseases, West German Centre of Infectious Diseases, University Hospital Essen, University Essen-Duisburg, 45147 Essen, Germany; lara.meller@uk-essen.de (L.M.); adalbert.krawczyk@uk-essen.de (A.K.); oliver.witzke@uk-essen.de (O.W.); 3 Department of Nephrology, University Hospital Essen, University of Duisburg-Essen, 45147 Essen, Germany; benjamin.wilde@uk-essen.de (B.W.); anja.gaeckler@uk-essen.de (A.G.)

**Keywords:** varicella–zoster virus, vaccination, ELISpot, kidney transplantation, sex dependency, diabetes mellitus

## Abstract

Solid organ transplant recipients have an up to ninefold higher risk of varicella–zoster virus (VZV) reactivation than the general population. Due to lifelong immunosuppressive therapy, vaccination against VZV may be less effective in kidney transplant (KTX) recipients. In the current study, twelve female and 17 male KTX recipients were vaccinated twice with the adjuvanted, recombinant zoster vaccine Shingrix™, which contains the VZV glycoprotein E (gE). Cellular immunity against various VZV antigens was analyzed with interferon-gamma ELISpot. We observed the strongest vaccination-induced changes after stimulation with a gE peptide pool. One month after the second vaccination, median responses were 8.0-fold higher than the responses prior to vaccination (*p* = 0.0006) and 4.8-fold higher than responses after the first vaccination (*p* = 0.0007). After the second vaccination, we observed an at least twofold increase in ELISpot responses towards gE peptides in 22 out of 29 patients (76%). Male sex, good kidney function, early time point after transplantation, and treatment with tacrolimus or mycophenolate were correlated significantly with higher VZV-specific cellular immunity, whereas diabetes mellitus was correlated with impaired responses. Thus, our data indicate that vaccination with Shingrix™ significantly augmented cellular, VZV gE-specific immunity in KTX recipients, which was dependent on several covariates.

## 1. Introduction

Varicella–zoster virus (VZV) is a member of the herpesvirus family that causes varicella/chickenpox after primary infection and zoster/shingles after reactivation. Viral DNA persists in neurons of the dorsal root and cranial nerve ganglia, where it can remain quiescent for decades [1]. As all herpesviruses, VZV may reactivate, especially in older and immunocompromised individuals [2,3]. Waning of VZV-specific cellular immunity is an important factor for VZV reactivation, and the age-dependent increase in shingles is correlated with the decrease in specific T cell immunity [4]. The incidence of shingles was up to ninefold higher in immunosuppressed solid organ transplant recipients than in the general population [5,6]. VZV causes a vesicular exanthema affecting one to three adjoining dermatomes, where it can lead to pain and postherpetic neuralgia [1,7].

In Germany, the United States, and many other countries, a live attenuated vaccine is licensed, and its use is recommended for vaccination against primary infection [8,9]. Moreover, to prevent reactivations, the use of a recombinant, adjuvanted VZV glycoprotein E (Shingrix^TM^, GlaxoSmithKline Biologicals S.A., Rixensart, Belgien) is recommended, especially from the age of 60 and for individuals with immunodeficiency [8]. This recombinant zoster vaccine contains an adjuvant based on liposomes, which serves as an amplifier of immunity [1]. Previous data indicate that vaccination with Shingrix^TM^ could reduce the risk of contracting shingles during one’s lifetime in the general population from 33% to 3% [10]. Moreover, data in kidney transplant (KTX) recipients indicate that it is also effective and may cut the rate of shingles by about half [5]. Considering 130 patients who received Shingrix^TM^ and 130 who received a placebo, a study by Vink et al. [5] reported a lower rate of suspected cases of shingles in vaccinees (3 vs. 7 suspected cases).

In the present study, we report on 29 KTX recipients who were vaccinated twice with Shingrix^TM^, in which VZV-specific cellular immunity was monitored at four time points prior to and post vaccination. We stimulated the patient cells with peptides of glycoprotein E (gE), the most abundant and immune-dominant glycoprotein of VZV [11], with a native VZV glycoprotein and with an inactivated whole VZV antigen. Immunity against VZV (gE) was measured with a highly sensitive interferon (IFN)-γ ELISpot assay, which detects specific T cells on a single-cell level [12]. Moreover, we compared responses in the patients with healthy controls and analyzed if covariates, such as sex, age, number of kidney transplantations, kidney function, co-morbidities, prior shingles, immunosuppressive therapy, allograft rejection, and interval between transplantation and vaccination or between vaccination and testing, had an impact on VZV-specific immunity.

## 2. Materials and Methods

### 2.1. Volunteers

Our prospective single-center study includes 29 KTX recipients who were tested longitudinally before and after vaccination with Shingrix^TM^. The participants in this observational study were recruited at the University Hospital Essen (Germany) in August 2020 according to the inclusion and exclusion criteria outlined in Table 1. The patient cohort contained twelve females and 17 males, and the median age at the time of the first blood sampling was 61 years (range: 45–79). The estimated glomerular filtration rate (eGFR, MDRD equation) [13] remained constant after vaccination (median values of 46–51 mL/min/1.73 m^2^). All patients reported previous chickenpox, and eight reported shingles. Ten patients were grafted with a living donor and 19 with a deceased donor. The patients were tested at the times of the first and second vaccination and approximately one and four months after the second vaccination. The median interval between the transplantation and first vaccination was 7.2 years, and that between the two vaccinations was 71 days.

In parallel, in August 2020, we included four age-matched, healthy controls (median age: 62 years, range: 60–65, three males and one female). All volunteers reported previous chickenpox, and the female reported previous shingles. According to the current recommendations [8], healthy individuals should be vaccinated against shingles from the age of 60, which defined the minimum age. Of note, none of the controls received immunosuppressive treatment. The median interval between their two vaccinations was 67 days.

The study was conducted according to the guidelines of the Declaration of Helsinki and was approved by the Ethics Committee of the University Hospital Essen, Germany (19-8700-BO, 18.12.2019). Informed consent was obtained from all subjects involved in the study.

### 2.2. Vaccine

The subunit vaccine Shingrix^TM^ contains 50 µg of the adjuvanted, recombinant VZV gE antigen produced in immortalized ovarian cells of the Chinese hamster (CHO cells) [14]. It is adjuvanted with AS01B containing 50 µg of the *Quillaja saponaria* Molina plant extract, fraction 21 (QS-21), and 50 µg 3-O-desacyl-4′-monophosphoryl lipid A (MPL) from *Salmonella minnesota*. Shingrix^TM^ is licensed for the prevention of shingles and postherpetic neuralgia in adults ≥50 years of age [14]. Vaccination consisted of two 0.5 mL doses injected into the deltoid muscle.

### 2.3. ELISpot Assay

Nine milliliters of heparinized blood was collected, and peripheral blood mononuclear cells (PBMCs) were separated through Ficoll gradient centrifugation. Numbers of PBMCs were determined with an automated hematology analyzer (XP-300, Sysmex, Norderstett, Germany). To assess VZV-specific cellular immunity, we performed IFN-γ ELISpot assays while using a peptide pool and two protein antigens as stimuli. In parallel experiments, we applied a gE peptide pool (1 µg/mL per peptide, JPT Peptide Technologies, Berlin, Germany), a native VZV glycoprotein (10 µg/mL, SERION), and a whole native VZV antigen (10 g/mL, SERION, Würzburg, Germany). The gE peptide pool contained 153 peptides derived from a peptide scan (15-mers with 11 aa overlap) through the envelope protein (Swiss-Prot ID: P09259) of the VZV strain Dumas. For the production of the two native antigens, VZV glycoprotein, and whole VZV antigen, HEL 299 cells were infected with the VZV strain Ellen. After cultivation, the antigens were isolated through lectin affinity chromatography or ultra-centrifugation through a sucrose cushion, respectively. The production of IFN-γ was determined using pre-coated ELISpot plates and a standardized detection system (T-Track^®^ ELISpot kit, Mikrogen GmbH, Neuried, Germany; formerly Lophius Biosciences GmbH, Regensburg, Germany). Cultures of 200,000 freshly isolated PBMCs were incubated without and with VZV antigens in 150 µL of AIMV medium (Gibco, Grand Island, USA) at 37 °C for 19 h. Stimulation with the T-cell mitogen phytohemagglutinin (PHA, 4 µg/mL) served as positive control. Colorimetric detection of cytokine-secreting cells was performed according to the manufacturer’s instructions. Spot numbers were analyzed with an ELISpot plate reader (AID Fluorospot, Autoimmun Diagnostika GmbH, Strassberg, Germany). VZV-specific spots were determined as stimulated minus non-stimulated (background) values (spot increment). Of note, the negative controls reached a median value of 0, a mean of 0.11 spots, and a standard deviation of 0.61 spots. The positive control with PHA indicated that all results included in this study were valid (median: 378 spot increment, range: 46–565).

### 2.4. Parameters with Potential Influence on Vaccination Responses

We considered age, kidney function (eGFR), interval between transplantation and first vaccination, interval between first and second vaccination, and interval between second vaccination and blood sampling as numerical variables. Moreover, sex, first vs. second kidney transplantation, living vs. deceased donor, diabetes mellitus, hypertension, coronary heart disease, previous malignant tumor, chronic obstructive pulmonary disease, previous cytomegalovirus, herpes simplex virus or VZV infection (chickenpox or shingles), previous antiviral treatment (acyclovir, valganciclovir, entecavir, cytotect), immunosuppressive therapy (tacrolimus, mycophenolate, corticosteroids, everolimus, azathioprine, ciclosporin, belatacept), and allograft rejections (total) were considered as categorical, dichotomous variables (yes/no).

### 2.5. Statistical Analysis

Data were analyzed using GraphPad Prism 8.4.2.679 (GraphPad Prism Software, San Diego, CA, USA) or IBM SPSS Statistics version 25 (Armonk, NY, USA). The calculation of the sample size was performed with the program G*Power 3.1.9.4 [15] using the following input parameters: one tail, an effect size of 0.55, an α error probability of 0.05, and a power (1-β error probability) of 0.95. This calculation yielded a total sample size of 27. The effect size was assumed based on preliminary data from a previous study [16]. Time courses of ELISpot responses were analyzed by using one-way ANOVA with Tukey’s multiple-comparison test. The results in transplant patients and healthy controls were compared by using a Mann–Whitney *U*-test. Correlation analyses of numerical variables were performed by Spearman test (two-tailed). The impact of categorical variables was also analyzed with the Mann–Whitney test. The impact of clinical variables on ELISpot responses was furthermore tested with multivariate analysis (multinomial logistic regression). If not otherwise stated, median values are indicated. Results were considered significant at *p* < 0.05.

## 3. Results

### 3.1. Time Course of ELISpot Responses to Three Different VZV Antigens

**Figure 1:**
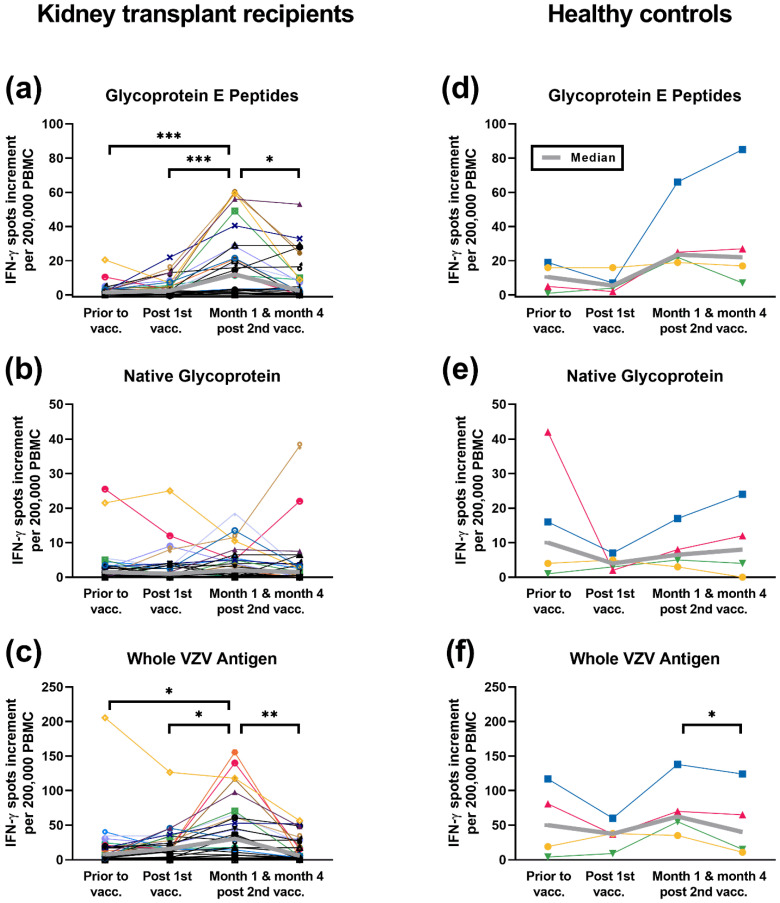
Time course of ELISpot responses towards various varicella–zoster virus (VZV) antigens in 29 kidney transplant recipients (**a**–**c**) and in four healthy controls (**d**–**f**). We used a peptide pool of glycoprotein E (**a**,**d**), a native glycoprotein (**b**,**e**), or a whole VZV antigen (**c**,**f**) for in vitro stimulation of peripheral blood mononuclear cells (PBMCs). Data prior to and post vaccination (vacc.) with Shingrix™ were compared by using one-way ANOVA with Tukey’s multiple-comparison test (* *p* < 0.05, ** *p* < 0.01, *** *p* < 0.001). VZV-specific spots were determined as stimulated minus non-stimulated (background) values (spot increment). The data for each individual is coded by the same color and symbol used consistently in panels (**a**) to (**c**) or (**d**) to (**f**). The bold gray line connects the median values.

In 29 KTX patients vaccinated with Shingrix™ (Table 2), we followed up the T cell responses towards a gE peptide pool, a native glycoprotein of VZV, and a whole VZV antigen (Table 3, Figure 1a–c). We observed the strongest vaccination-induced changes after stimulation with the gE peptide pool. One month after the second vaccination, median responses were 8.0-fold higher than the responses prior to vaccination (*p* = 0.0006) and 4.8-fold higher than the responses after the first vaccination (*p* = 0.0007). However, at month 4 vs. 1 after the second vaccination, ELISpot responses already declined significantly (*p* = 0.01) (Figure 1a). The results on the native glycoprotein showed a similar trend, i.e., a maximum response at month 1 after the second vaccination and, thereafter, a decrease in ELISpot responses (Figure 1b). After stimulation with the whole VZV antigen, vaccination-induced changes also reached statistical significance (Figure 1c). One month after the second vaccination, median responses were 4.1-fold higher than the responses prior to vaccination (*p* = 0.03) and 1.9-fold higher than the responses after the first vaccination (*p* = 0.01).

The ELISpot responses in the four vaccinated healthy controls (Figure 1d–f) were overall higher than those of the KTX recipients, reaching statistical significance (*p* < 0.05) for stimulation with the gE peptide pool at month 4 after the second vaccination (Table 3). Overall, there was a greater drop in immunity in the patients than in the healthy controls. Thus, we could detect significant VZV (gE)-specific cellular responses in vaccinated KTX recipients, and the gE peptide pool, which is the immunogenic component of the subunit vaccine Shingrix™, appeared to be the best stimulus for assessing VZV (gE)-specific cellular vaccination responses.

Moreover, we calculated how many patients showed an at least twofold increase in ELISpot responses at month 1 after the second vaccination vs. baseline. After stimulation with the gE peptide pool, 22 out of 29 patients (76%) fulfilled this criterion, which we used to assess the response rate for cell-mediated immunity. The respective number for the native glycoprotein was 6 out of 29 (21%), and for the whole VZV antigen, it was 17 out of 29 (59%).

### 3.2. Correlation of VZV-Specific Cellular Immunity with Clinical Parameters

**Figure 2:**
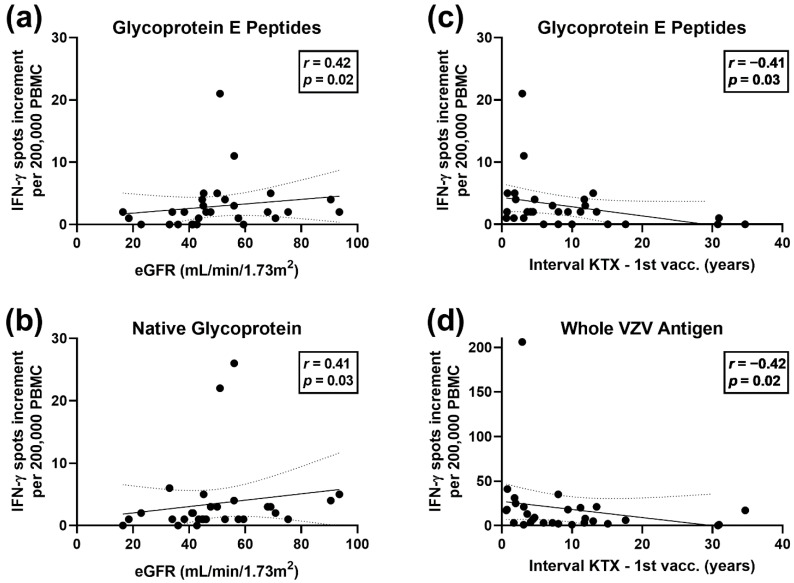
Spearman correlation analysis of estimated glomerular filtration rate (eGFR) or interval between transplantation and first vaccination and ELISpot responses prior to vaccination. In 29 kidney transplant recipients, we observed a positive correlation of eGFR and ELISpot responses towards a peptide pool of glycoprotein E (**a**) and towards the native glycoprotein (**b**). The correlation was negative between the interval between transplantation and first vaccination and ELISpot responses towards a peptide pool of glycoprotein E (**c**), as well as towards whole varicella–zoster virus (VZV) (**d**). The continuous line represents the regression line, and the broken lines represent the 95% confidence interval.

With a univariate analysis, we determined if ELISpot responses were correlated with patients’ characteristics, as outlined in Section 2.4. A Spearman analysis of the numerical variables indicated that the eGFR prior to vaccination was correlated positively with the ELISpot responses to the gE peptide pool (*r* = 0.42 and *p* = 0.02) and to the native glycoprotein of VZV (*r* = 0.41 and *p* = 0.03), i.e., patients with a better kidney function showed higher VZV (gE)-specific ELISpot responses at baseline (Figure 2a,b). After vaccination, however, the correlation was no longer significant.

Moreover, the interval between transplantation and first vaccination was correlated negatively with baseline ELISpot responses to the gE peptide pool (*r* = −0.41 and *p* = 0.03) and to the whole VZV antigen (*r* = −0.42 and *p* = 0.02) (Figure 2c,d). Thus, patients tested early after transplantation showed higher VZV-specific cellular responses.

**Figure 3:**
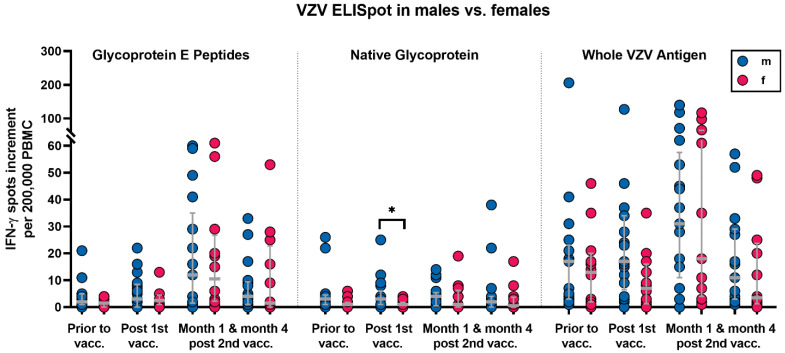
Varicella–zoster-virus-specific ELISpot responses in 17 male and twelve female kidney transplant recipients prior to and after the first and second vaccination with Shingrix™. Blue dots indicate males and red dots indicate females. VZV-specific spots were determined as stimulated minus non-stimulated (background) values (spot increment). Gray horizontal lines represent median values and the interquartile range. Data were compared by using a Mann–Whitney test (* *p* < 0.05).

**Figure 4:**
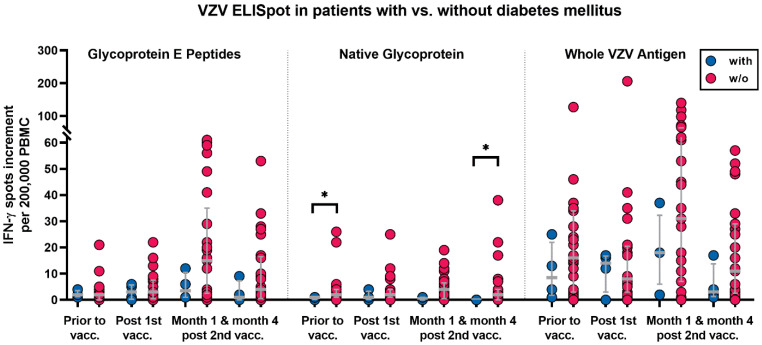
Varicella–zoster-virus-specific ELISpot responses in kidney transplant recipients with and without diabetes mellitus prior to and after the first and second vaccination with Shingrix™. Blue dots indicate four patients with diabetes mellitus (with) and red dots indicate 25 patients without (w/o). VZV-specific spots were determined as stimulated minus non-stimulated (background) values (spot increment). Gray horizontal lines represent median values and the interquartile range. Data were compared by using a Mann–Whitney test (* *p* < 0.05).

The analysis of categorical variables could identify male sex, diabetes mellitus, and treatment with tacrolimus and mycophenolate as factors influencing the cellular VZV-specific immunity. In detail, males vs. females showed stronger VZV-specific responses, which reached statistical significance for responses towards the native glycoprotein after the first vaccination (*p* = 0.03) (Figure 3). Diabetic patients had weaker cellular responses, which were significant for stimulation with the native glycoprotein prior to vaccination and at month 4 after the second vaccination (*p* = 0.04 and *p* = 0.02, respectively) (Figure 4).

Patients treated with tacrolimus had stronger ELISpot responses after the second vaccination, reaching significance for the gE peptide pool at month 1 (*p* = 0.02) and for the whole VZV antigen at month 1 and month 4 (*p* = 0.03 and *p* = 0.04, respectively) (Table 4). Patients receiving mycophenolate had stronger ELISpot responses prior to vaccination and after the first and second vaccination (Table 4). The results were significant for the peptide pool, native glycoprotein, and whole VZV antigen prior to vaccination (*p* = 0.03, *p* = 0.03 and *p* = 0.002, respectively), for the whole VZV antigen after the first vaccination (*p* = 0.01), and for all three VZV antigens at month 4 after the second vaccination (*p* = 0.045, *p* = 0.03 and *p* = 0.006, respectively).

The remaining clinical parameters had no significant influence on VZV (gE)-specific cellular immunity. However, age tended to correlate negatively with ELISpot responses prior to and post vaccination, i.e., older patients had slightly lower ELISpot responses.

The correlation of the clinical parameters with significant results with the univariate analysis was further examined by using multivariate analysis (Table 5). The VZV (gE)-specific ELISpot results correlated significantly with kidney function (eGFR), with the interval between transplantation and first vaccination, and with sex, diabetes mellitus, and treatment with mycophenolate. For treatment with tacrolimus, only one significant correlation was found, which could also have arisen by chance. Considering long-term immunity (at month 4 after the second vaccination), the interval between transplantation and vaccination had the strongest impact on VZV gE-specific responses (χ^2^ = 54.0). Immunity towards the native glycoprotein at month 4 was similarly affected by eGFR, the interval to transplantation, and mycophenolate (χ^2^ = 39.7–44.4), and, to a lesser extent, by sex (χ^2^ = 28.4) and diabetes mellitus (χ^2^ = 22.9). Finally, immunity towards the whole VZV antigen at month 4 was especially affected by diabetes mellitus (χ^2^ = 937.3), followed by sex (χ^2^ = 58.9) and interval to transplantation (χ^2^ = 29.8).

### 3.3. Correlation of VZV-Specific Immunity Measured with Various VZV Antigens and at Various Time Points

**Figure 5:**
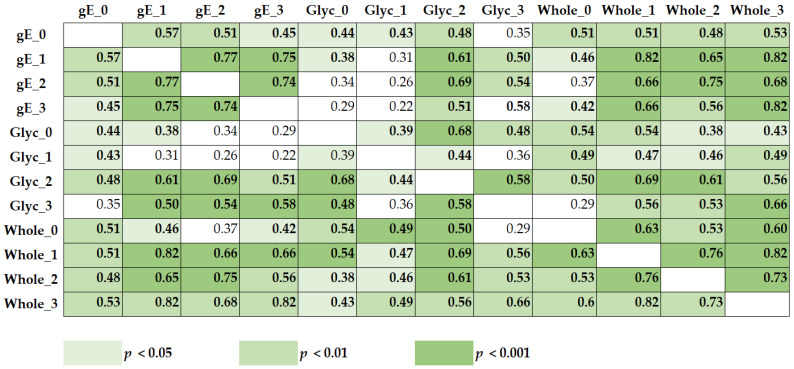
Spearman correlation of ELISpot responses towards a peptide pool of glycoprotein E (gE), a native glycoprotein (Glyc), and a whole varicella–zoster virus (Whole) in 29 kidney transplant recipients. Each patient was tested four times, i.e., prior to vaccination (0), after the first vaccination (1), at month 1 after the second vaccination (2), and at month 4 after the second vaccination (3). The numbers indicate the correlation coefficient *r*, which always showed a positive correlation (0.22–0.82). Significant correlations are highlighted in bold; the color indicates the level of significance.

The Spearman analysis in 29 KTX recipients showed that the ELISpot responses to the different VZV antigens and at the different time points were positively correlated, i.e., immunity to one VZV antigen was predictive of a response to the other two antigens, and data at the different time points were also correlated (Figure 5).

## 4. Discussion

The current data indicate that vaccination with two shots of Shingrix^TM^ could significantly increase VZV (gE)-specific cellular immunity in KTX recipients, which was detected after in vitro stimulation with a gE peptide pool and a whole VZV antigen. However, as compared to the healthy controls, the cellular responses were lower, as expected. A comparative analysis of various VZV antigens showed that vaccination-induced changes in VZV-specific immunity were most pronounced after stimulation with the gE peptide pool, where we observed an 8.0-fold increase after the second vaccination compared to the baseline. Similar results were observed in a cohort of hematopoietic stem cell transplant recipients, where the gE peptide pool was also most suitable for measuring VZV (gE)-specific vaccination responses [16]. As the zoster vaccine Shingrix^TM^ contains recombinant gE, the most abundant and immune-dominant glycoprotein expressed on the surface of VZV-infected cells [11], this finding appears plausible. It has been shown that gE is a major target for VZV-specific antibody responses [17]. Previously, a strong correlation of glycoprotein-specific antibodies and protection against varicella was shown [18]. In addition, IgG antibodies against gE and IgG antibodies against whole VZV showed positive correlations when analyzing the data qualitatively (positive/negative, 99% agreement) [19] and quantitatively (correlation coefficient of 0.86%) [20]. Similarly to these antibody data, we observed a significant correlation of cellular responses to gE and to whole VZV antigens. Previously, Cassaniti et al. showed that the ELISpot response after stimulation with gE peptides is mainly a CD4 T cell response [21]. This group measured immunity in (unvaccinated) kidney transplant recipients and found an overall range of ELISpot responses that was similar to what we observed in the current study.

There are already data on T cell immunity after vaccination with Shingrix™ in a cohort of 32 kidney transplant recipients [5]. However, immunity was determined through intracellular cytokine staining and detection was performed using flow cytometry after stimulation of CD4 T cells with a pool of peptides covering the gE ectodomain. This study showed a vaccine response rate for cell-mediated immunity of 71% at month 2, defined as an at least twofold increase in responses after two vaccinations. In the current study, we used another method to assess cellular immunity, we tested the samples at month 1 after the second vaccination, and we stimulated PBMCs and not CD4 T cells. Nevertheless, we applied the same criterion, i.e., we determined the percentage of patients with an at least twofold increase in responses after two vaccinations. After stimulation with the gE peptide pool, we found a response rate of 76%. Thus, the data generated by the two different methods fit well.

Moreover, vaccination with Shingrix™ had no effect on allograft function as defined by serum creatinine [5], which could be confirmed by our current data. The correlation of kidney function with immune function is well established [22,23], and therefore, a positive correlation of eGFR with VZV-specific cellular immunity prior to vaccination is in line with current knowledge.

The interval between transplantation and testing and ELISpot results showed a negative correlation, i.e., sooner after transplantation, cellular immune responses were higher. This observation was not expected at first glance. Especially within the first years after transplantation, reactivation of herpesviruses is common [24], and it can be speculated that (subclinical) reactivation caused by immunosuppression leads to an expansion of T cells directed against herpesviruses, such as VZV or cytomegalovirus (CMV). An increased frequency of these specific T cells may result in stronger VZV-specific ELISpot responses at baseline if it is closer to transplantation. This hypothesis is supported by the fact that we observed stronger cellular responses towards CMV in dialysis patients with vs. without immunosuppressive treatment [25] and a higher rate of CMV-specific proliferative responses in hematopoietic stem cell transplant recipients vs. healthy controls [26]. Another unexpected finding, the positive correlation of treatment with tacrolimus or mycophenolate and increased VZV-specific ELISpot responses, may have been caused by a similar phenomenon: (subclinical) VZV reactivation. However, as the majority of patients were treated with tacrolimus (86%), the observation needs to be interpreted with caution. Of note, two of the patients who did not receive tacrolimus were treated with belatacept and did not develop any cellular responses to vaccination. This finding is in accordance with recent data showing that patients who received belatacept also did not respond to vaccination against SARS-CoV-2 [27,28,29].

In addition, we could identify male sex as a factor correlated with increased VZV-specific immunity. Consistently with that finding, the previous literature indicated that the incidence of shingles also differed between males and females [6]. The annual rate per 1000 person-years was lower in males (2.6 vs. 3.8, *p* < 0.0001), which could be explained by stronger VZV-specific T cell immunity. Several studies showed sex-dependent immune responses—for example, various concentrations of cytokines or vaccine antibodies [23,30,31,32,33,34,35,36]. In females, cytomegalovirus pp65-specific IL-21 ELISpot responses were higher [23] or antibody titers after vaccination against hepatitis B or SARS-CoV-2 virus were increased [30,37]. However, males showed a trend of higher cellular responses towards pneumococcal antigens [38]. It is, therefore, quite possible that VZV-specific immunity is also sex-dependent.

The correlation of diabetes mellitus with impaired cellular responses was expected because hyperglycemia in diabetes is thought to cause dysfunction of the immune response, which fails to control the spread of invading pathogens and makes diabetic subjects more susceptible to infections [39]. We observed a trend of impaired cellular immune response for all VZV antigens and at almost all time points. Since our cohort contained only four patients with diabetes mellitus, this finding did not reach statistical significance for all comparisons.

## 5. Conclusions

In KTX recipients, vaccination with the adjuvanted, recombinant vaccine Shingrix™, which contains the VZV gE, led to a significant increase in in vitro cellular responses, especially towards VZV gE. This is the first study assessing vaccination efficacy in this setting with ELISpot, an assay that measures active secretion of IFN-γ upon stimulation with VZV antigens. However, as compared to age-matched controls, cellular immune responses after vaccination were weaker in kidney transplant recipients. Furthermore, we could identify sex, kidney function, time point after transplantation, immunosuppressive drugs, and diabetes mellitus as covariates of VZV (gE)-specific cellular vaccination responses; these have not yet been reported.

## Figures and Tables

**Table 1:** Inclusion and exclusion criteria.

Inclusion	Exclusion
Age ≥ 45 years	Acute rejection ^2^
Interval to kidney transplantation ≥ 6 months	Active shingles infection
Interval to shingles ≥ 2 months	Acute (other) infection (fever > 38.5 °C)
Stable kidney function ^1^	Actual malignant tumor
Complete clinical dataset	Allergy against a component of the vaccine
Sequential ELISpot data at four time points	Pregnancy
Written informed consent	Inability to consent

^1^ Estimated glomerular filtration rate of >15 mL/min/1.73 m^2^ and change in serum creatinine of <1.5-fold within the month prior to inclusion; ^2^ defined by change in serum creatinine of >20% within one month prior to inclusion.

**Table 2:** Characteristics of the 29 kidney transplant recipients tested prior to and post vaccination with Shingrix™.

Variable	Group	Absolute Number or Median (Range)
**Sex**	Female	12
	Male	17
Age (years)		61 (45–79)
Kidney transplantation, no.	First	24
	Second	5
eGFR	Prior to vacc.	46 (16–94)
(mL/min/1.73 m^2^)	Post 1st vacc.	49 (12–99)
	M1 post 2nd vacc.	51 (14–94)
	M4 post 2nd vacc.	47 (15–88)
Co-morbidities	Diabetes mellitus	4
	Hypertension	12
	Coronary heart disease	8
	Previous malignant tumor	11
	COPD	4
Anamnesis of	Cytomegalovirus	11
previous infection	Herpes simplex virus type 1	2
with herpesviruses	VZV (chickenpox)	29
	VZV (shingles)	8
Previous	Aciclovir	1
antiviral treatment	Valganciclovir	3
	Entecavir	1
	Cytotect	1
Immunosuppressive	Tacrolimus	25
therapy	Mycophenolate	20
	Corticosteroids	26
	Everolimus	5
	Azathioprine	1
	Ciclosporin	1
	Belatacept	2
Allograft rejection	Total	6
	Acute	5
	Acute and chronic	1
	Humoral	2
	Cellular	3
	Humoral and Cellular	1
Interval transplantation– 1st vaccination		7.2 years (8 months–34.7 years)
Interval 1st vaccination– 2nd vaccination		71 days (62–149)
Interval 2nd vaccination– blood sampling		
	First follow-up	1.2 months (0.9–1.9)
	Second follow-up	4.2 months (3.7–9.6)

eGFR—estimated glomerular filtration rate; vacc.—vaccination with Shingrix™; COPD—chronic obstructive pulmonary disease; VZV—varicella–zoster virus.

**Table 3:** Comparison of varicella–zoster virus (VZV)-specific ELISpot responses in 29 kidney transplant (KTX) recipients and four healthy controls (HC).

Antigen	Time Point	KTX			HC			*p*
		Median	MIN	MAX	Median	MIN	MAX	
Glycoprotein E Peptides	Pre vacc.	**1.5**	−0.5	20.5	**10.5**	1	19	0.07
	post 1st vacc.	**2.5**	−1	22	**5.5**	2	16	0.11
	M1 post 2nd vacc.	**12**	0	60.5	**23.5**	19	66	0.09
	M4 post 2nd vacc.	**2.5**	0	53	**22**	7	85	0.04 *
Native Glycoprotein	Pre vacc.	**1.5**	0	25.5	**10**	1	42	0.10
	post 1st vacc.	**1**	0	25	**4**	2	7	0.15
	M1 post 2nd vacc.	**2**	0	18.5	**6.5**	3	17	0.09
	M4 post 2nd vacc.	**1.5**	0	38	**8**	0	24	0.17
Whole VZV Antigen	Pre vacc.	**7.5**	0	205.5	**50**	4	117	0.10
	post 1st vacc.	**16**	0	126.5	**37.5**	9	60	0.09
	M1 post 2nd vacc.	**30.5**	0	155.5	**62.5**	35	138	0.08
	M4 post 2nd vacc.	**6**	0	56.5	**40**	11	124	0.07

VZV—specific cellular immunity is indicated as the spot increment, i.e., stimulated vs. non-stimulated (background) values. Median values are highlighted in bold. MIN—minimum; MAX—maximum; M—month; vacc.—vaccination with Shingrix™. Data were compared by using a Mann–Whitney test (* *p* < 0.05).

**Table 4:** Correlation of varicella–zoster virus (VZV)-specific ELISpot responses and immunosuppressive treatment in 29 kidney transplant recipients.

Variable	Antigen	Time Point	Treatment Received			Treatment Not Received			*p*
			Median	MIN	MAX	Median	MIN	MAX	
**Tacrolimus**	Glycoprotein E Peptides	Pre vacc.	**2**	0	21	**1.5**	0	2	0.32
		post 1st vacc.	**3**	0	22	**1**	0	2	0.06
		M1 post 2nd vacc.	**15**	0	61	**1.5**	0	3	0.02 *
		M4 post 2nd vacc.	**5**	0	53	**0.5**	0	1	0.05
	Native Glycoprotein	Pre vacc.	**2**	0	26	**1.5**	1	3	0.74
		post 1st vacc.	**2**	0	25	**1**	0	4	0.34
		M1 post 2nd vacc.	**4**	0	19	**0.5**	0	2	0.12
		M4 post 2nd vacc.	**2**	0	38	**0**	0	1	0.06
	Whole VZV Antigen	Pre vacc.	**8**	0	206	**9.5**	1	21	0.55
		post 1st vacc.	**16**	0	127	**6**	2	17	0.21
		M1 post 2nd vacc.	**35**	0	140	**7**	1	11	0.03 *
		M4 post 2nd vacc.	**12**	0	57	**2**	1	3	0.04 *
**Mycophenolate**	Glycoprotein E Peptides	Pre vacc.	**2**	0	21	**0**	0	4	0.03 *
		post 1st vacc.	**3**	0	22	**1**	0	8	0.06
		M1 post 2nd vacc.	**13.5**	0	60	**3**	0	61	0.33
		M4 post 2nd vacc.	**8.5**	0	53	**0**	0	25	0.045 *
	Native Glycoprotein	Pre vacc.	**2.5**	1	26	**1**	0	4	0.03 *
		post 1st vacc.	**2.5**	0	25	**1**	0	4	0.21
		M1 post 2nd vacc.	**4**	0	19	**1**	0	14	0.08
		M4 post 2nd vacc.	**3**	0	38	**0**	0	3	0.03 *
	Whole VZV Antigen	Pre vacc.	**17.5**	1	206	**3**	0	9	0.002 *
		post 1st vacc.	**19**	0	127	**3**	0	46	0.01 *
		M1 post 2nd vacc.	**36**	1	140	**18**	0	117	0.24
		M4 post 2nd vacc.	**16.5**	2	57	**1**	0	20	0.006 *

Median values are highlighted in bold. MIN—minimum; MAX—maximum; M—month; vacc.—vaccination with Shingrix™. Data were compared by using a Mann–Whitney test (* *p* < 0.05).

**Table 5:** Multivariate analysis of varicella–zoster virus (VZV)-specific ELISpot responses and clinical parameters in 29 kidney transplant recipients.

Antigen	Time Point	eGFR	Interval to KTX ^1^	Sex	Diabetes Mellitus	Tacrolimus	Mycophenolate
Glycoprotein E Peptides	Pre vacc.	<0.0001	0.002		<0.0001		<0.0001
	post 1st vacc.						
	M1 post 2nd vacc.						<0.0001
	M4 post 2nd vacc.		<0.0001				
Native Glycoprotein	Pre vacc.	0.02	0.01	<0.0001			0.02
	post 1st vacc.		0.046				
	M1 post 2nd vacc.	<0.0001		<0.0001			
	M4 post 2nd vacc.	<0.0001	<0.0001	0.0001	0.006		<0.0001
Whole VZV Antigen	Pre vacc.	0.01	0.02				0.001
	post 1st vacc.	0.003	<0.0001		<0.0001		
	M1 post 2nd vacc.	<0.0001	<0.0001	<0.0001	<0.0001	<0.0001	0.01
	M4 post 2nd vacc.		0.04	<0.0001	<0.0001		

^1^ Interval between kidney transplantation (KTX) and first vaccination (vacc.) with Shingrix™. Data were compared by using multinomial logistic regression, and significant *p* values are indicated. eGFR—estimated glomerular filtration rate.

## Data Availability

The data presented in this study are available on request from the corresponding author. The data are not publicly available due to privacy restrictions.
